# Mapping the Galvanic Corrosion of Three Metals Coupled with a Wire Beam Electrode: The Influence of Temperature and Relative Geometrical Position

**DOI:** 10.3390/ma11030357

**Published:** 2018-02-28

**Authors:** Hong Ju, Yuan-Feng Yang, Yun-Fei Liu, Shu-Fa Liu, Jin-Zhuo Duan, Yan Li

**Affiliations:** 1 College of Mechanical and Electronic Engineering, China University of Petroleum, Qingdao 266580, China; 1209lyf@sina.com (Y.-F.L.); 17864269818@163.com (S.-F.L.); yuangungun_1204@163.com (J.-Z.D.); yanlee@upc.edu.cn (Y.L.); 2 Corrosion and Protection Centre, The University of Manchester, Manchester M13 9PL, UK; Yuanfeng.Yang@manchester.ac.uk

**Keywords:** desalination, galvanic corrosion, wire-beam electrode, heterogeneous electrochemistry, temperature, relative geometrical position

## Abstract

The local electrochemical properties of galvanic corrosion for three coupled metals in a desalination plant were investigated with three wire-beam electrodes as wire sensors: aluminum brass (HAl77-2), titanium (TA2), and 316L stainless steel (316L SS). These electrodes were used with artificial seawater at different temperatures. The potential and current–density distributions of the three-metal coupled system are inhomogeneous. The HAl77-2 wire anodes were corroded in the three-metal coupled system. The TA2 wires acted as cathodes and were protected; the 316L SS wires acted as secondary cathodes. The temperature and electrode arrangement have important effects on the galvanic corrosion of the three-metal coupled system. The corrosion current of the HAl77-2 increased with temperature indicating enhanced anode corrosion at higher temperature. In addition, the corrosion of HAl77-2 was more significant when the HAl77-2 wires were located in the middle of the coupled system than with the other two metal arrangement styles.

## 1. Introduction

Water and energy shortages plague many communities around the world [1,2,3,4,5]. More than 1.2 billion people lack access to clean and safe drinking water [1,4]. This water shortage will likely increase in the coming decades. Therefore, much effort has been focused on desalination. Various desalination technologies—both thermally-driven and membrane-based—have been increasingly used to convert seawater to fresh water.

Multi-effect desalination (MED) is one of the most common techniques [6]. A growing number of industrial plants have performed this type of thermal desalination in recent years [6,7,8]. It offers lower capital requirements and costs, simple operation/maintenance, higher thermal efficiency, higher heat-transfer coefficients, lower energy consumption, and higher performance ratios [7,8]. The key evaporators of MED technologies are made of aluminum brass, titanium, and stainless steel. They must offer long-term stability even with corrosive seawater. Indeed, distillation equipment is susceptible to salt corrosion at high temperature [9]; galvanic corrosion is also inevitable in these metals and manifests gradually over the lifetime of a desalination plant. Galvanic corrosion easily induces and accelerates other types of localized corrosion and can pose a serious threat to the safe operation of the desalination plants. Somewhat surprisingly, there is little documentation on multi-metal galvanic corrosion—especially in desalination plants. Therefore, a comprehensive study into the behavior and mechanism of multi-metal galvanic corrosion—particularly the smallest variations in electrochemical signals—remains an important study area.

Galvanic corrosion is an enhanced corrosion between two or more kinds of electrically connected metals [10] in which the more active metal acts as the anode, and the less active metal is the cathode [11]. Galvanic corrosion is common in municipal infrastructure and industry [12] and is a common research topic. The galvanic corrosion rate and the potential distribution over a galvanic couple depend on the electrochemical properties of the metals; environmental variables such as temperature, salinity, oxygen content, and solution flow; and the geometry of the corroding system [13]. Therefore, determining the correlation between electrochemical parameters underlying galvanic corrosion and the corrosion factors is important to clarifying the behavior and mechanism of the galvanic corrosion.

To date, several established electrochemical techniques have been used for this purpose. However, the electrochemical processes of galvanic corrosion are usually inhomogeneous. Time-honored electrochemical techniques that use a single conventional electrode are limited to determining these localized corrosion processes. To overcome this problem, multi-electrodes (also known as wire-beam electrode (WBE)) were first developed in 1991 by Tan [14,15]. The WBE has been used in corrosion research for several years [16,17,18,19,20,21,22]. The remarkable feature of this method is that potential/current distributions can be measured under complex surface conditions [23]. Each wire in a WBE is an individual electrochemical sensor. This enables a WBE to measure electrochemical parameters from local areas of the electrode surface via wires located in these areas. Therefore, the electrode array could be used equally well to investigate the galvanic corrosion of couples composed of different metals or alloys.

This work will investigate the influence of temperature and geometrical arrangement for mapping the galvanic corrosion of three coupled metals in desalination plant using an advanced WBE method. The distribution of both the potential and the current density with different temperatures and metal arrangements of electrode arrays were obtained. This provides valuable information on the localized corrosion process. This work studied multiple metals and their galvanic corrosion in desalination plants. It also provides important theoretical values with practical significance for the structural design of safe future desalination devices.

## 2. Materials and Methods

### 2.1. WBE Fabrication

**Figure 1:**
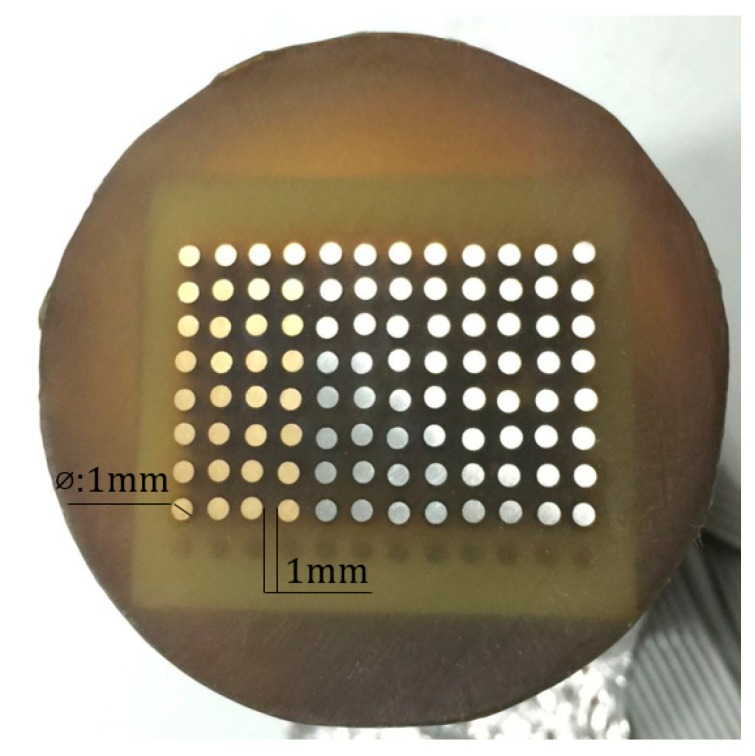
Schematic of a wire-beam electrode.

The WBE was fabricated from 96 wires arranged in an 8 × 12 matrix (1-mm diameter). These were embedded in an epoxy resin at 1-mm intervals (Figure 1). The 96 microelectrodes in the WBE were fabricated from aluminum brass (HAl77-2), 316L stainless steel (316L SS), and titanium (TA2). The area of the 96 microelectrodes was approximately 0.75 cm^2^. The chemical composition of the real samples (HAl77-2, 316L SS, and TA2) are given in Table 1.

**Figure 2:**
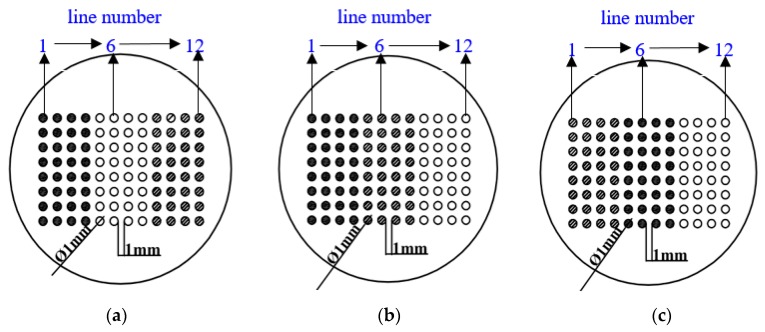
Schematic of the three WBEs with different metal arrangement sequences: (**a**) WBE1, HAl77-2/316L SS/TA2; (**b**) WBE2, HAl-77-2/TA2/316L SS; and (**c**) WBE3, TA2/HAl77-2/316L SS (● *Aluminum-brass* (*HAl77-2*); ○ *Titanium* (*TA2*); Ø *Stainless steel* (*316L SS*)).

Three different WBEs were used (Figure 2): WBE1 (HAl77-2/316L SS/TA2); WBE2 (HAl77-2/TA2/316L SS); and WBE3 (TA2/HAl77-2/316L SS). The surface of the WBE was polished with increasingly fine sandpaper (500- to 1200-grit). It was then cleaned with acetone and deionized water.

### 2.2. Experimental Conditions

WBE experiments used artificial seawater at varying temperatures. The concentrations of Cl^−^ were 1.5 wt % or 2.7 wt %. The constituents were NaCl (19.46 g/L, Shanghai, China), MgCl_2_·6H_2_O (8.80 g/L, Shanghai, China), Na_2_SO_4_ (3.25 g/L, Shanghai, China), and CaCl_2_ (0.92 g/L, Shanghai, China) with a Cl^−^ concentration of 1.5 wt %. The second formulation was NaCl (35.26 g/L), MgCl_2_·6H_2_O (16.03 g/L), Na_2_SO_4_ (5.80 g/L), and CaCl_2_ (1.67 g/L) for a Cl^−^ concentration of 2.7 wt %. The artificial seawater was prepared with ultrapure water and A.R. reagents in accordance with the national standard. The experiments were conducted at 30, 40, 50, 60, and 70 °C.

Open-circuit potential (OCP), local potential, and local current were measured by WBE technology [12,17,22]. The equipment consisted of a chassis (PXI-1033, NI™, Austin, TX, USA), a 7.5-digit digital multimeter (PXI-4071, NI™, Austin, TX, USA), an effective guard/current amplifier (PXI-4022, NI™, Austin, TX, USA), and a high-density FET switch matrix module (PXI-2535, NI™, Austin, TX, USA). The performance metrics of these components have been reported in detail [12,17,22].

All wire sensors were disconnected for approximately 30 min to monitor their open circuit potential (OCP) values. After reaching a stable OCP, the WBEs were short-circuited for 12 h, and the local potential and current measurements were collected separately. Each WBE electrode wire was temporarily separated from the other connected electrodes to record the local potential in the sequence against a saturated calomel electrode (SCE). These were then reconnected to the other electrodes. Individual wire sensors short-circuited the electrodes described above. These were first separated and connected to a device to record the local current; all other wires were shorted together. These processes were controlled by a computer and via self-designed software, in a LabVIEW environment. Finally, the potential and local current distributions were mapped with Surfer 8.0 software (Golden, CO, USA) [22]. Each experiment was repeated over three parallel runs with unique samples to verify the accuracy of the experiment.

## 3. Results and Discussion

### 3.1. Effect of Temperature on the Corrosion Behavior of Three-Metal Coupled Systems

The temperature-effect investigation on the corrosion behavior of the three-metal coupled systems was performed in artificial seawater with a chloride ion concentration of 2.7 wt %.

#### 3.1.1. Corrosion Behavior of WBE1 in Artificial Seawater at Different Temperatures

**Figure 3:**
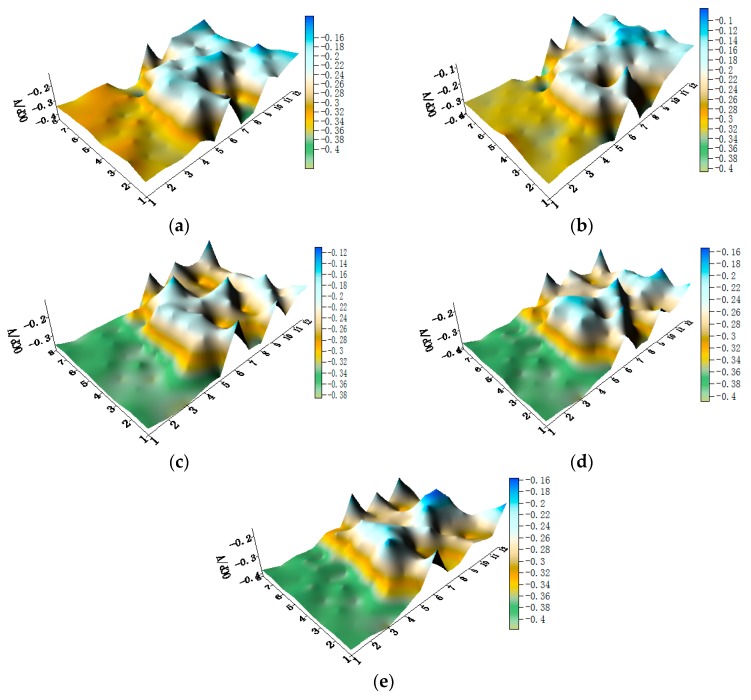
Open circuit potential OCP (vs. saturated calomel electrode (SCE)/V) distribution maps of the WBE1 (HAl77-2/316L SS/TA2) after immersion in artificial seawater at different temperatures: (**a**) 30 °C; (**b**) 40 °C; (**c**) 50 °C; (**d**) 60 °C; and (**e**) 70 °C.

OCP distribution maps of the WBE1 after immersion in artificial seawater at different temperatures are shown in Figure 3 (the 2D maps also are shown in Figure S1 of the Supplementary Materials). The OCP value of HAl77-2 was the most negative of the three metals. The OCP values of 316L SS and TA2 were similar and were both higher than HAl77-2. The HAl77-2 was an anode, and 316L SS and TA2 were cathodes in the three-metal coupled system.

**Figure 4:**
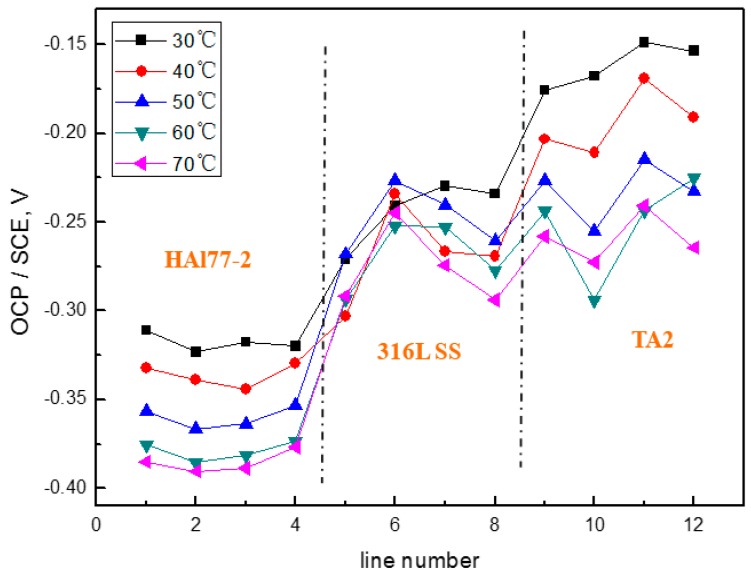
Average OCP distribution of each microelectrode line for WBE1 after immersion in artificial seawater at different temperatures.

The average OCP distribution of each microelectrode line for WBE1 after immersion in artificial seawater at different temperatures is presented in Figure 4. Figure 4 shows the OCP values of HAl77-2, 316L SS, and TA2 had constant negative shifts with increasing temperature. This suggests that the tendency of corrosion in metals increases with temperature because the mass-transfer rate increased with temperature; the diffusion of dissolved oxygen also accelerated with temperature.

**Figure 5:**
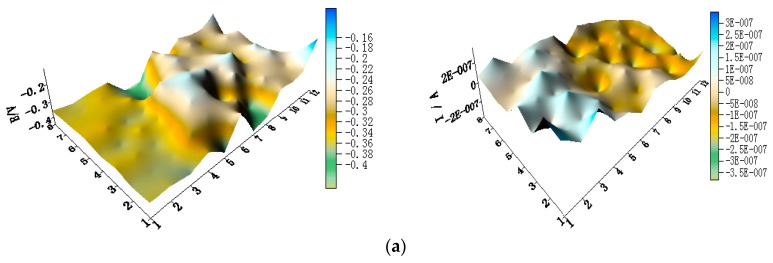

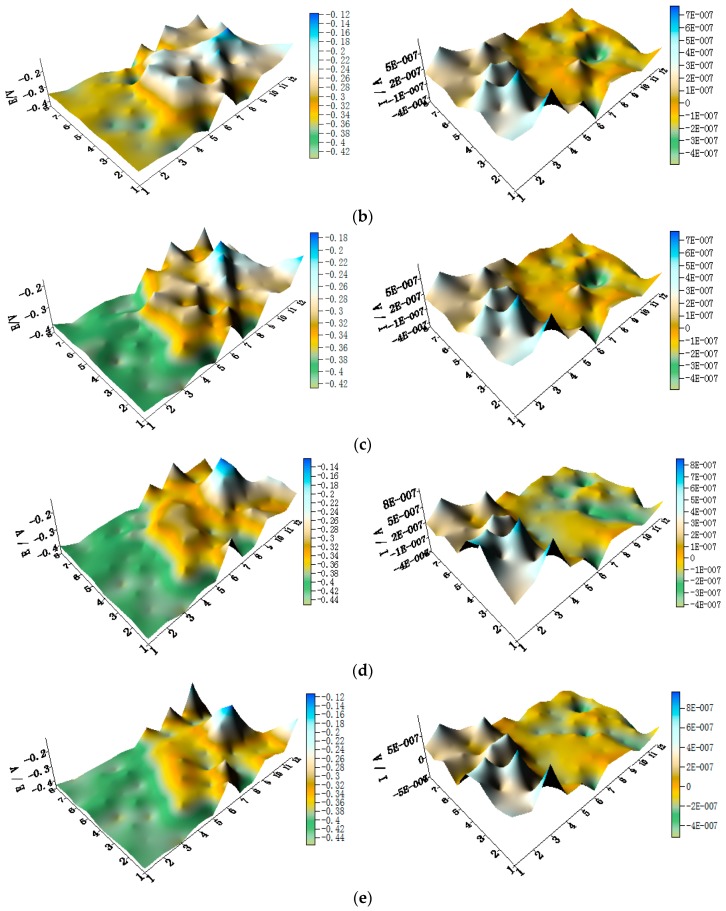
Spatial potential (**left**, E vs. SCE/V) and current–density (**right**, I/A) distribution maps of WBE1 after being short-circuited for 12 h in artificial seawater at different temperatures: (**a**) 30 °C; (**b**) 40 °C; (**c**) 50 °C; (**d**) 60 °C; and (**e**) 70 °C.

Figure 5 shows the spatial potential and current–density distribution maps of WBE1 after being short-circuited for 12 h in artificial seawater at different temperatures (the 2D maps also are shown in Figure S2 of the Supplementary Materials). The data indicate that the potential of HAl77-2 is lowest. Its corresponding local current density is the most positive of the three metals. Therefore, HAl77-2 is a corrosion anode in WBE1. The potential of TA2 is the most positive of the three metals. Its corresponding current density is negative. Thus, TA2 is the cathode in the coupling system. The local potential value of 316L SS is between HAl77-2 and TA2. Most 316L SS wires had negative local currents; only a few 316L SS wires had positive local currents. Therefore, some of the 316L SS wires were cathodes, and others were local anodes.

**Figure 6:**
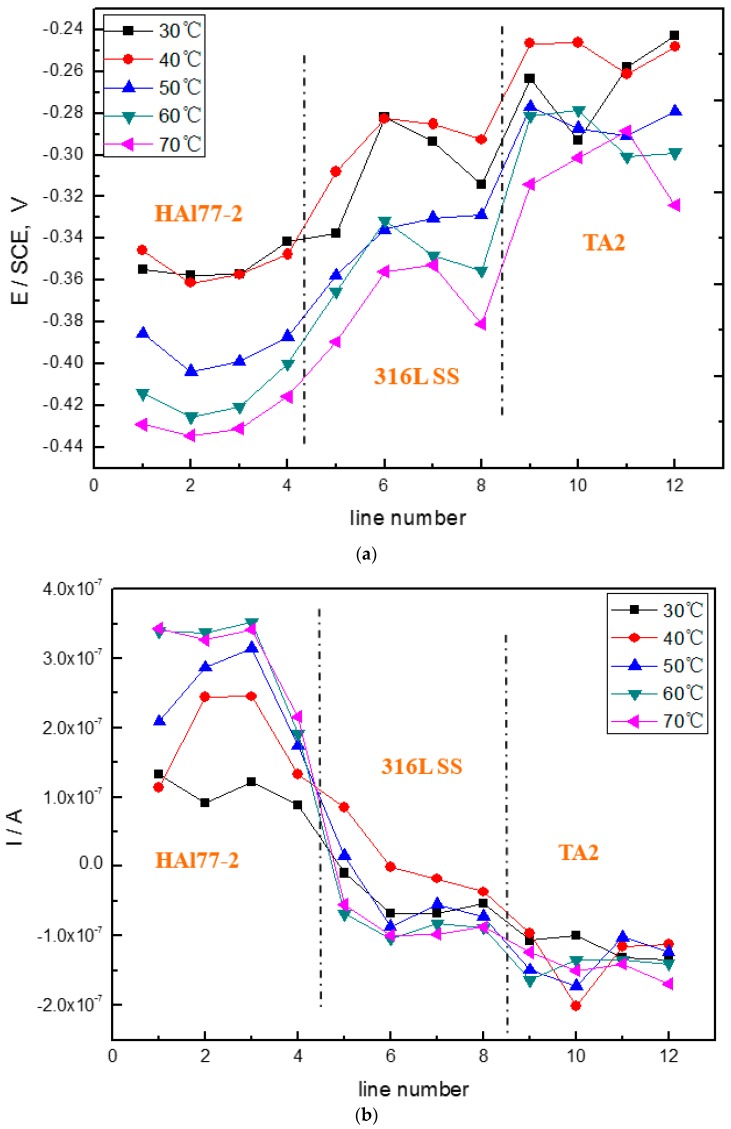
(**a**) Average potential; and (**b**) current–density distributions of each WBE1 microelectrode line after being short-circuited for 12 h in artificial seawater at different temperatures.

Figure 6 shows the average potential and current–density distributions of each WBE1 microelectrode line after being short-circuited for 12 h in artificial seawater at different temperatures. The local potential of the three metals drops with increasing temperature. The local current of HAl77-2 increases with temperature indicating that the anode corrosion is of greater significance when the temperature of the solution increases.

#### 3.1.2. Corrosion Behavior of WBE2 in Artificial Seawater at Different Temperatures

**Figure 7:**
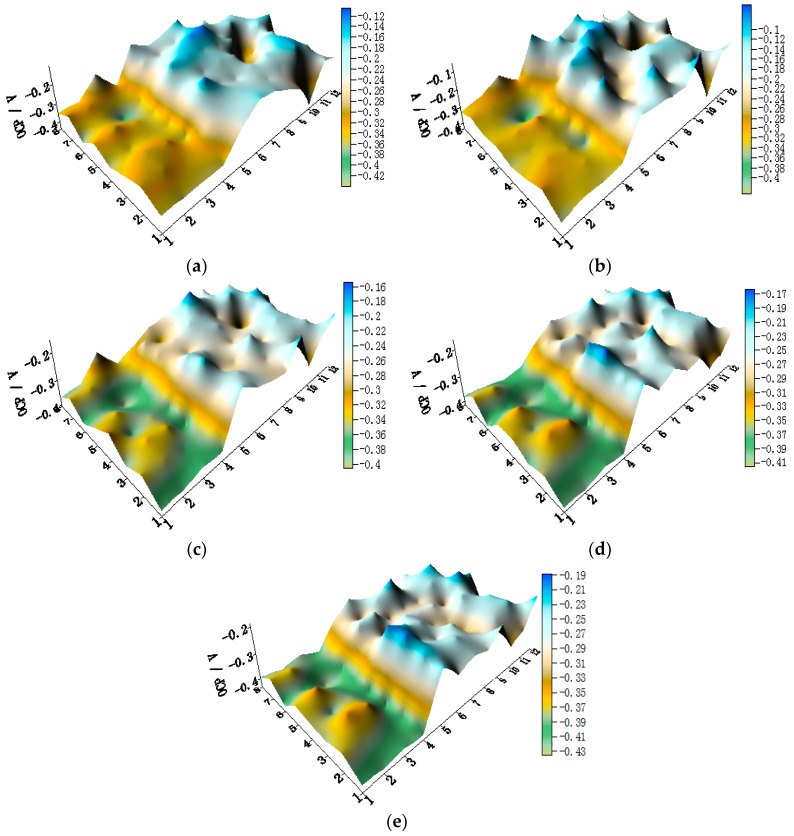
OCP (vs. SCE/V) distribution maps of the WBE2 after immersion in artificial seawater at different temperatures: (**a**) 30 °C; (**b**) 40 °C; (**c**) 50 °C; (**d**) 60 °C; and (**e**) 70 °C.

Figure 7 shows OCP distribution maps of the WBE2 after immersion in artificial seawater at different temperatures. The OCP value of HAl77-2 is the most negative of the three metals. The OCP values of 316L SS and TA2 are similar, and both are higher than HAl77-2. Therefore, HAl77-2 results in anodic corrosion, and 316L SS and TA2 are cathodes in the three-metal coupled system in artificial seawater. In addition, the OCP distribution of HAl77-2 is homogeneous, and the OCP distribution of 316L SS and TA2 fluctuant because the passive film on the metal surface is damaged by the chloride ions. These results are similar to WBE1 findings.

**Figure 8:**
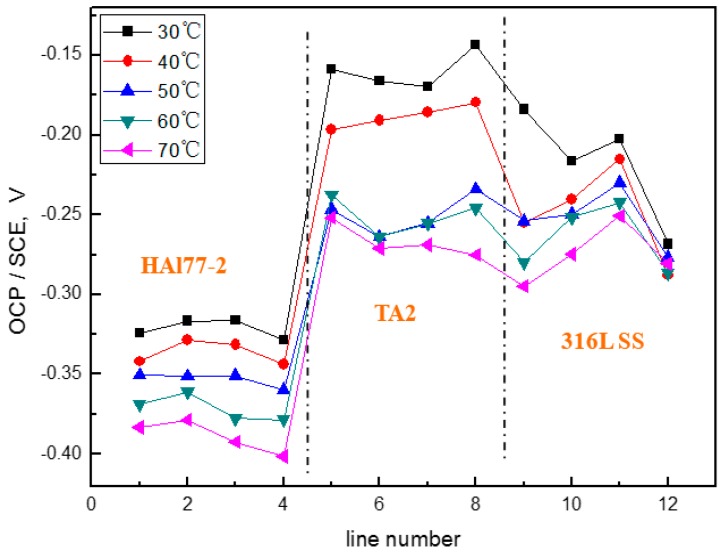
Average OCP distribution of each microelectrode line for WBE2 after immersion in artificial seawater at different temperatures.

Figure 8 shows the average OCP distribution of each microelectrode line for WBE2 after immersion in artificial seawater at different temperatures. Figure 8 also shows the OCP values of HAl77-2; both 316L SS and TA2 shift toward a negative potential.

**Figure 9:**
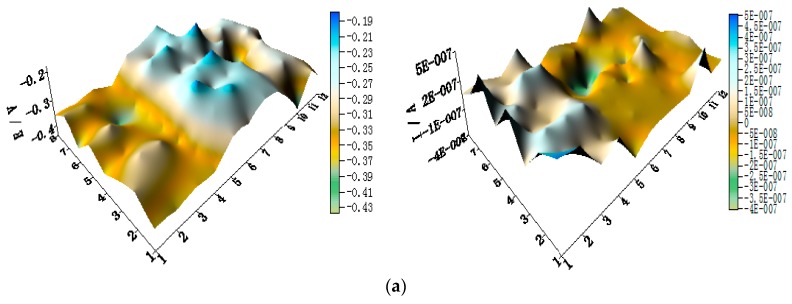

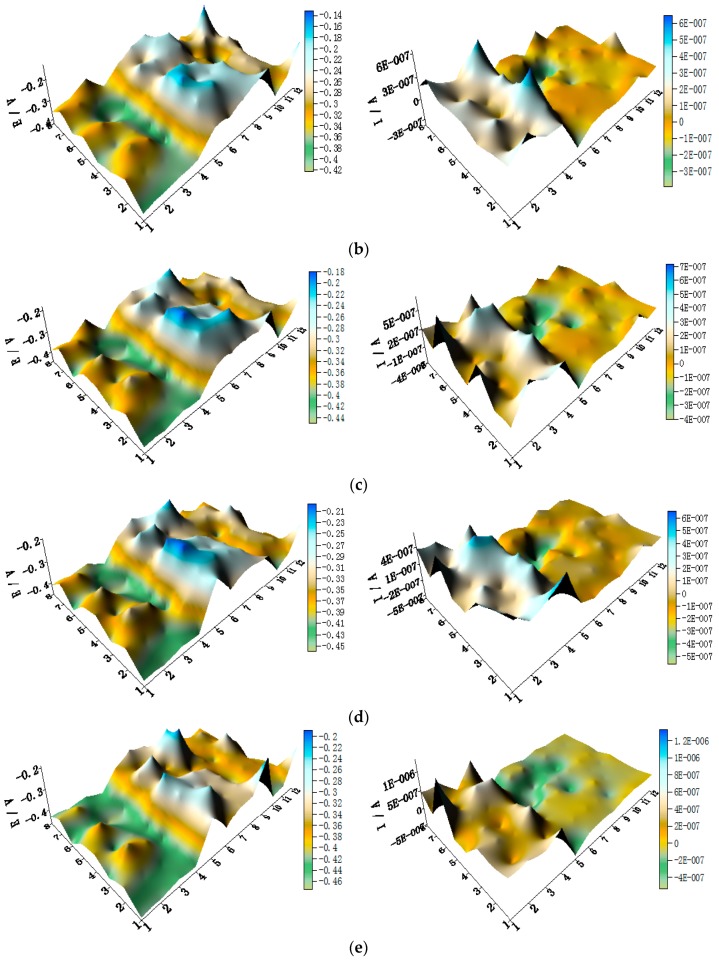
Spatial potential (**left**, E vs. SCE/V) and current–density (**right**, I/A) distribution maps of WBE2 after being short-circuited for 12 h in artificial seawater at different temperatures: (**a**) 30 °C; (**b**) 40 °C; (**c**) 50 °C; (**d**) 60 °C; and (**e**) 70 °C.

Figure 9 shows spatial potential and current–density distribution maps of WBE2 after being short-circuited for 12 h in artificial seawater at different temperatures. The potential of HAl77-2 is the most negative of the three metals. Its current density is positive indicating that HAl77-2 has anode corrosion in WBE2. TA2 is the cathode because of its positive potential and negative current values. The potential of 316L SS is between HAl77-2 and TA2, but the current performance of most 316L SS wires is negative. This reveals that 316L SS is the secondary cathode in WBE2.

**Figure 10:**
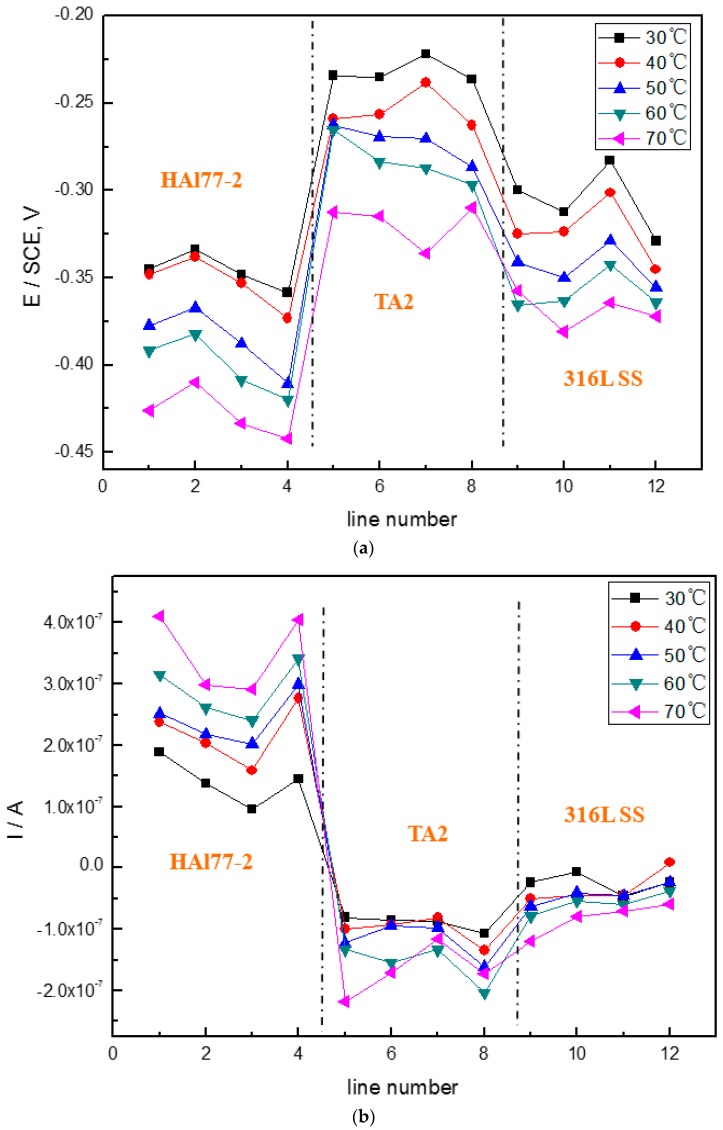
(**a**) Average potential and (**b**) current–density distributions of each WBE2 microelectrode line after being short-circuited for 12 h in artificial seawater at different temperatures.

Figure 10 shows the average potential and current–density distributions of each WBE2 microelectrode line after being short-circuited for 12 h in artificial seawater at different temperatures. The potentials of the three metals obviously have a negative shift with increasing temperature. The positive local current of HAl77-2 increases with temperature, whereas the negative local current of 316L SS and TA2 decreases with temperature. There is more anode and galvanic corrosion at higher temperature.

#### 3.1.3. Corrosion Behavior of WBE3 in Artificial Seawater at Different Temperatures

**Figure 11:**
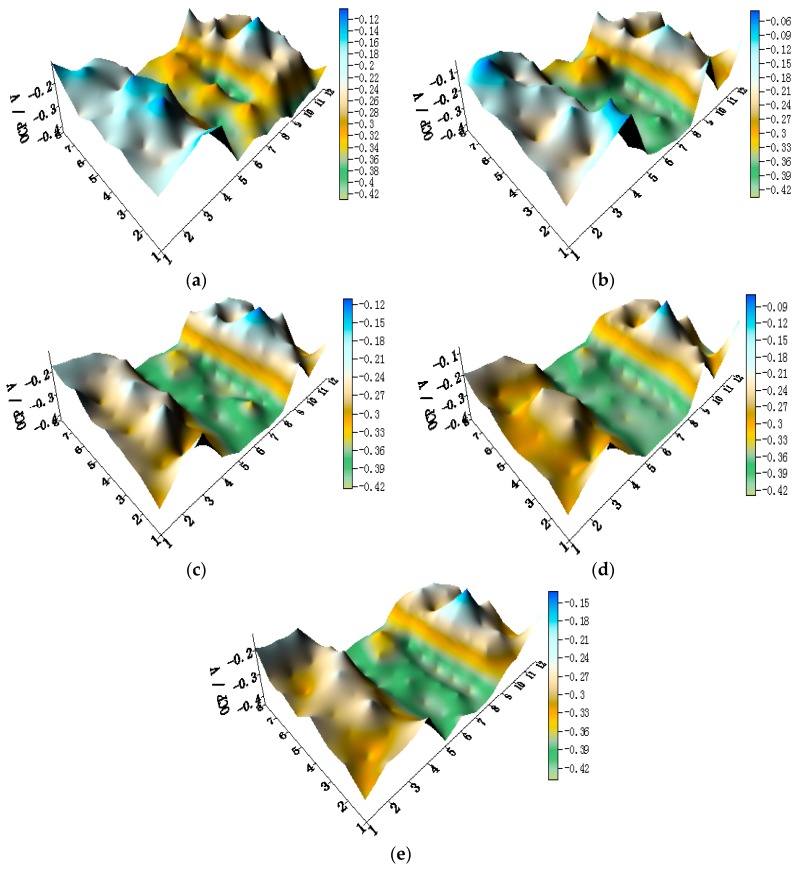
OCP (vs. SCE/V) distribution maps of the WBE3 after immersion in artificial seawater at different temperatures: (**a**) 30 °C; (**b**) 40 °C; (**c**) 50 °C; (**d**) 60 °C; and (**e**) 70 °C.

Figure 11 shows OCP distribution maps of the WBE3 after immersion in artificial seawater at different temperatures. Similar to WBE2 and WBE3, HAl77-2 has the most negative OCP value; the OCP values of 316L SS and TA2 are higher than HAl77-2.

**Figure 12:**
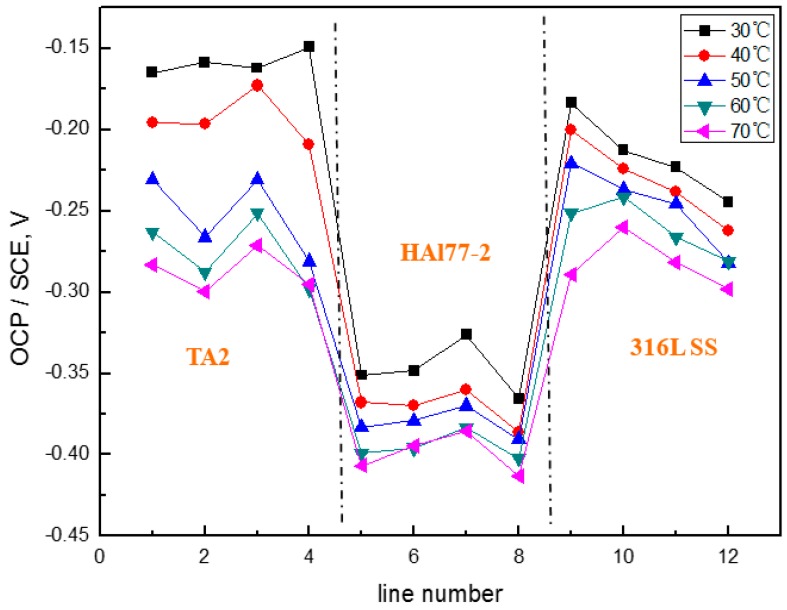
Average OCP distribution of each microelectrode line for WBE3 after immersion in artificial seawater at different temperatures.

Figure 12 shows the average local potential and current–density distribution of each WBE3 microelectrode line after being short-circuited for 12 h in artificial seawater at different temperatures. The OCPs of HAl77-2, 316L SS, and TA2 shifted negatively with increases in temperature because the reaction activity enhances with increasing temperature.

**Figure 13:**
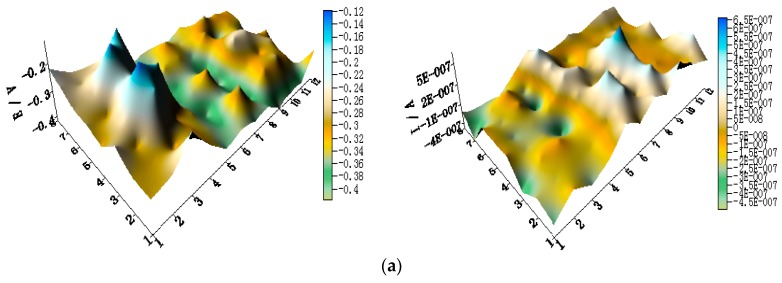

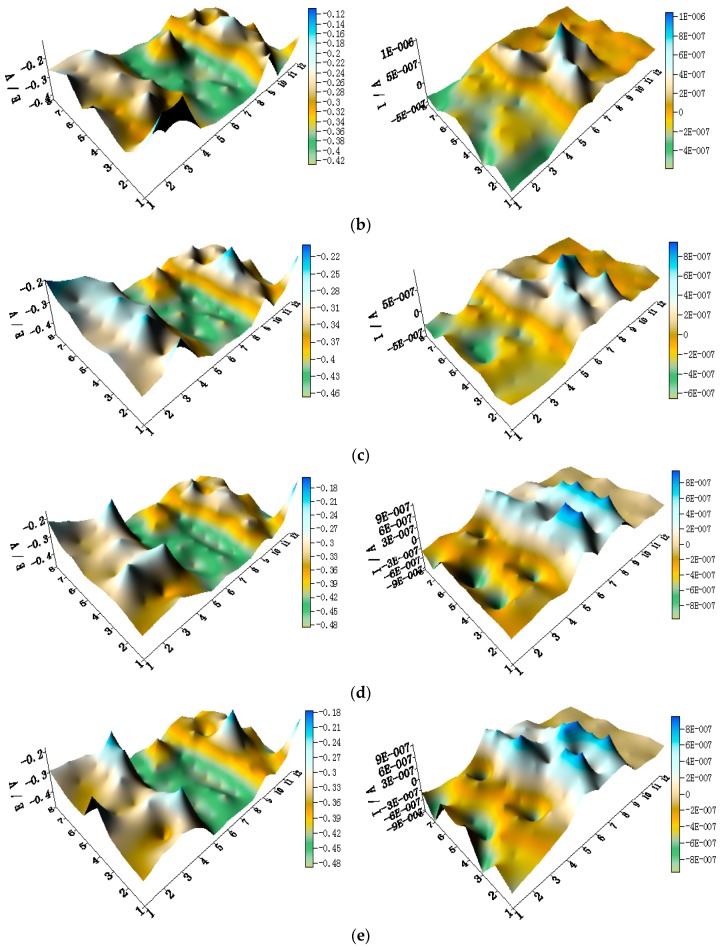
Spatial local potential (**left**, E vs. SCE/V) and current–density (**right**, I/A) distribution maps of WBE3 after being short-circuited for 12 h in artificial seawater at different temperatures: (**a**) 30 °C; (**b**) 40 °C; (**c**) 50 °C; (**d**) 60 °C; and (**e**) 70 °C.

Figure 13 shows spatial potential and current–density distribution maps of WBE3 after being short-circuited for 12 h in artificial seawater at different temperatures. HAl77-2 has the most negative local potential in WBE3; its corresponding local current is positive indicating that HAl77-2 behaves as the anode in WBE3. The TA2 has the highest local potential in WBE3, but its corresponding local current is negative suggesting that TA2 is a cathode in WBE3. The local potential of 316L SS is higher than that of HAl77-2 but is slightly lower than TA2 indicating that 316L SS is the secondary cathode. These results are similar to the findings for WBE1 and WBE2.

**Figure 14:**
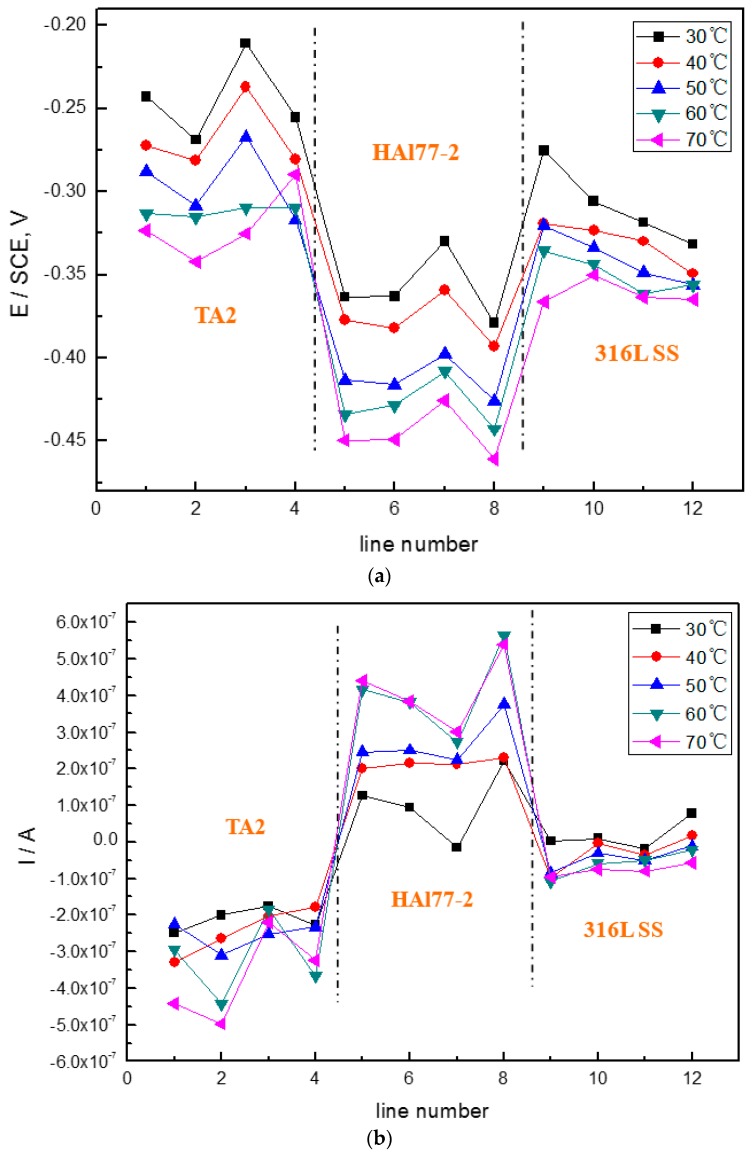
(**a**) Average local potential; and (**b**) current–density distributions of each WBE3 microelectrode line after being short-circuited for 12 h in artificial seawater at different temperatures.

Figure 14 shows the average local potential and current–density distributions of each WBE3 microelectrode line after being short-circuited for 12 h in artificial seawater at different temperatures. The local potential of the three metals shift negative with increasing temperature. The local current of HAl77-2 increases with temperature indicating that corrosion becomes more prominent at higher temperatures.

### 3.2. Effect of the Relative Position of Metal on the Corrosion Behavior of Three Metal-Coupled Systems

#### 3.2.1. Corrosion Behavior of Three Coupled Systems in Artificial Seawater: Cl^−^ 1.5 wt % and 30 °C

**Figure 15:**
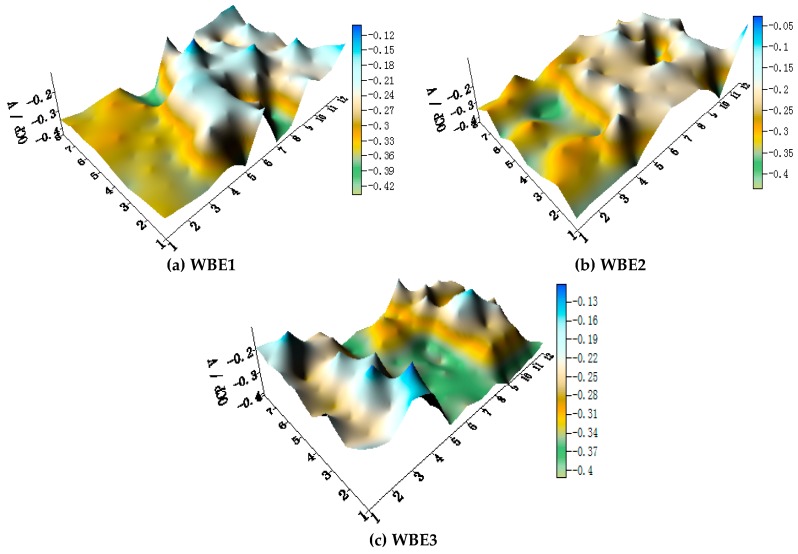
OCP (vs. SCE/V) distribution maps of three coupled electrodes after immersion in artificial seawater: 1.5% (Cl^−^, wt %) and 30 °C: (**a**) WBE1, HAl77-2/316L SS/TA2; (**b**) WBE2, HAl-77-2/TA2/316L SS; and (**c**) WBE3, TA2/HAl77-2/316L SS.

The OCP distributions of three coupled electrodes after immersion in artificial seawater with 1.5 wt % Cl^−^ at 30 °C are shown in Figure 15. Regardless of the arrangement of the three metals, the OCP of HAl77-2 is always the most negative, followed by 316L SS; the OCP of TA2 is always the highest.

**Figure 16:**
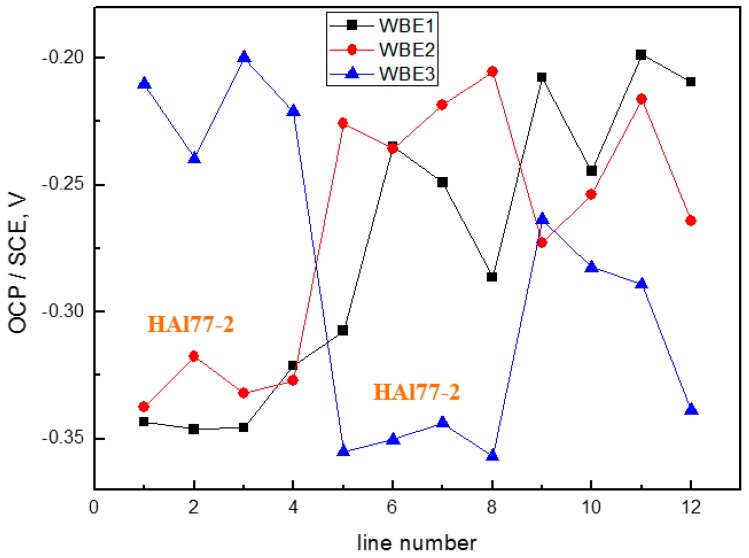
Average OCP distribution of each microelectrode line for the three coupled electrodes after immersion in artificial seawater with 1.5 wt % Cl^−^ at 30 °C.

Figure 16 shows the average OCP distribution of each microelectrode line for the three coupled electrodes after immersion in artificial seawater with 1.5 wt % Cl^−^ at 30 °C. The OCP values of HAl77-2 in WBE3 are lower than those in WBE1 and WBE2. The potential of 316L SS was between the values of the HAl77-2 and the TA2, accompanied by numerical fluctuations. In addition, the OCP distribution maps of 316L SS and TA2 were more inhomogeneous than those of HAl77-2, possibly because of the effect of chloride ions on the passivation films of 316L SS and TA2 in artificial seawater.

**Figure 17:**
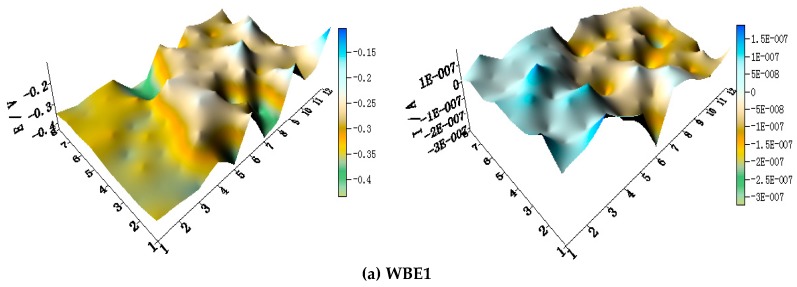

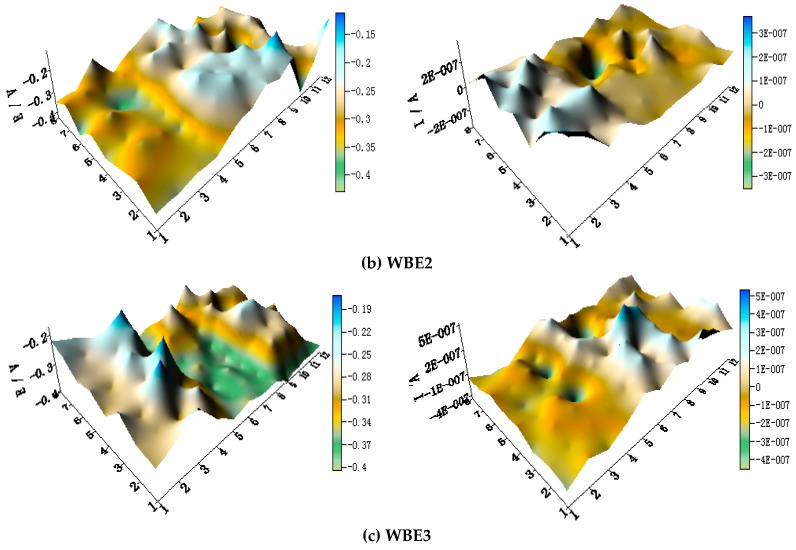
Spatial potential (**left**, E vs. SCE/V) and current–density (**right**, I/A) distribution maps of three coupled electrodes after being short-circuited for 12 h in artificial seawater with 1.5 wt % Cl^−^ at 30 °C: (**a**) WBE1, HAl77-2/316L SS/TA2; (**b**) WBE2, HAl-77-2/TA2/316L SS; and (**c**) WBE3, TA2/HAl77-2/316L SS.

**Figure 18:**
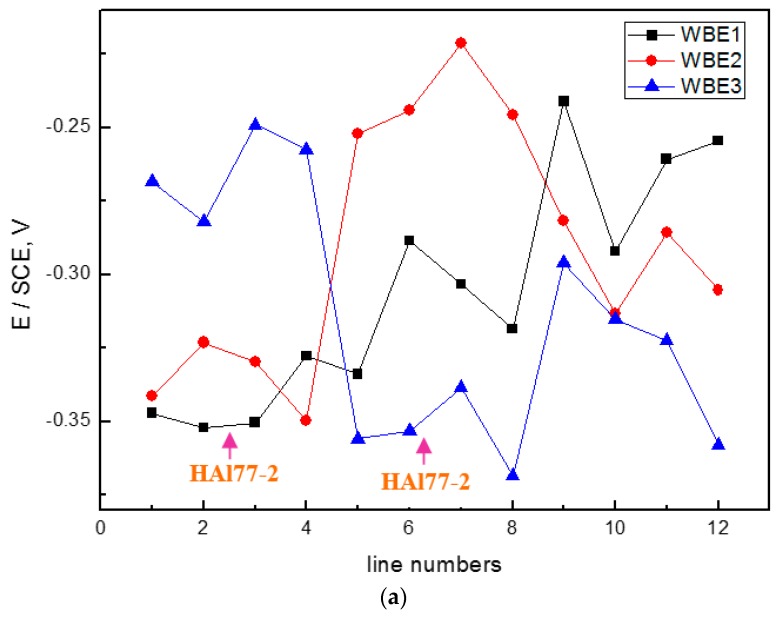

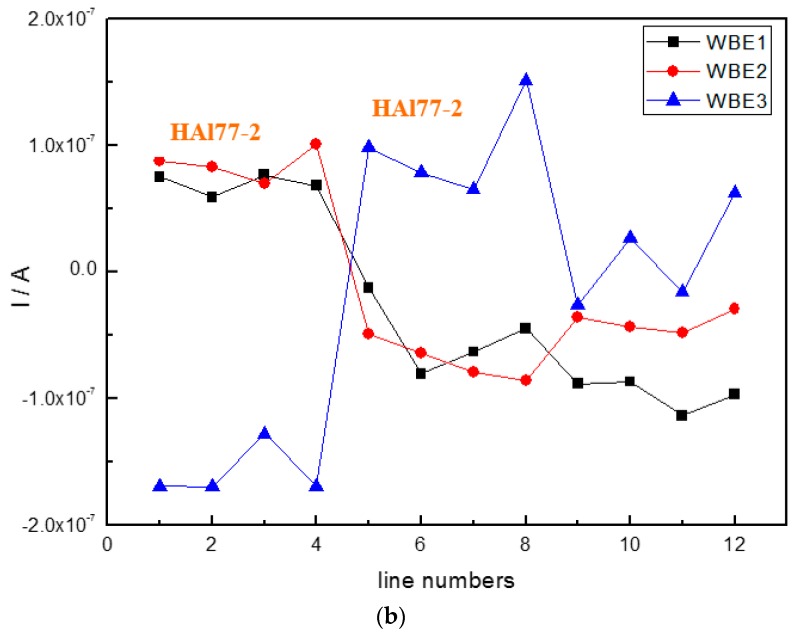
(**a**) Average potential; and (**b**) current–density distributions of each microelectrode line after being short-circuited for 12 h in artificial seawater with 1.5 wt % Cl^−^ at 30 °C.

Figure 17 shows the spatial potential and current–density distribution maps of the three coupled electrodes after being short-circuited for 12 h in artificial seawater with 1.5 wt % Cl^−^ at 30 °C. The findings in Figure 17 suggest that the local potential of HAl77-2 is always the most negative regardless of the sequence of the coupled systems. The local current remains positive indicating that HAl77-2 is the anode in all three coupled systems. Figure 18 presents the average potential and current–density distributions of each microelectrode line after being short-circuited for 12 h in artificial seawater with 1.5 wt % Cl^−^ at 30 °C. The potential of HAl77-2 is the most negative when HAl77-2 is between TA2 and 316L SS in the WBE3. The potential of HAl77-2 is more positive when HAl77-2 is at the edge and next to the TA2 in WBE2. The average current of HAl77-2 in WBE3 behaves at the maximum value, and anode corrosion is most significant when HAl77-2 is in the middle of the coupled system. Moreover, the current values of the fifth and eighth lines of WBE3 were significantly higher than those of the sixth and seventh lines, which reveals that the corrosion at the interface between the anode and the cathode is more serious than that away from the interface.

#### 3.2.2. Corrosion Behavior of Three Coupled Systems in Artificial Seawater: Cl^−^ 2.7 wt % and 70 °C

**Figure 19:**
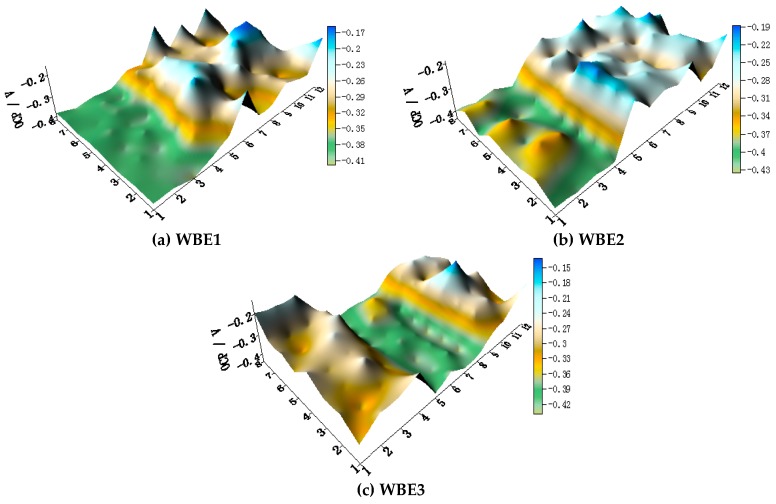
The OCP distribution maps of the three electrodes after immersion in artificial seawater with 2.7 wt % Cl^−^ at 70 °C: (**a**) WBE1, HAl77-2/316L SS/TA2; (**b**) WBE2, HAl-77-2/TA2/316L SS; and (**c**) WBE3, TA2/HAl77-2/316L SS.

Figure 19 shows the OCP distribution maps of the three electrodes (WBE1, WBE2, and WBE3) in artificial seawater with 2.7 wt % Cl^−^ at 70 °C. The OCP value of HAl77-2 is obviously always the most negative in WBE1, WBE2, and WBE3.

**Figure 20:**
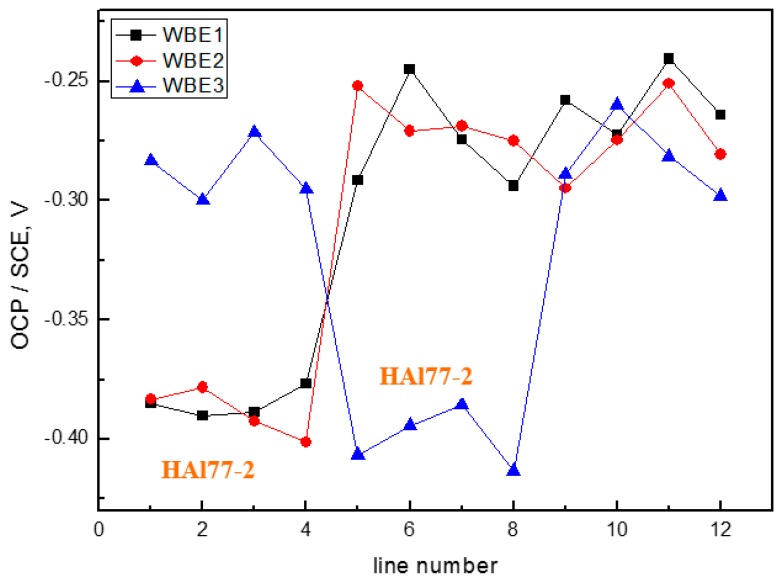
Average OCP distribution of each microelectrode line for the three electrodes after immersion in artificial seawater with 2.7 wt % Cl^−^ at 70 °C.

Figure 20 shows the average OCP distribution of each microelectrode line for the three electrodes after immersion in artificial seawater with 2.7 wt % Cl^−^ at 70 °C. The OCP value of HAl77-2 in WBE3 is lower than that in WBE1 and WBE2.

**Figure 21:**
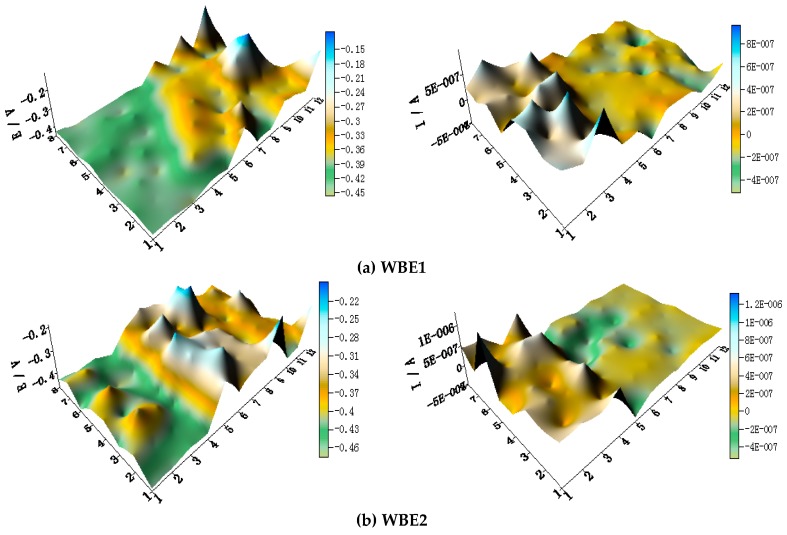

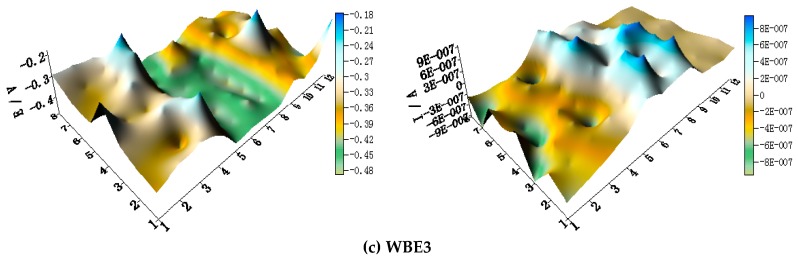
Spatial potential (**left**, E vs. SCE/V) and current–density (**right**, I/A) distribution maps of three electrodes after being short-circuited for 12 h in artificial seawater with 2.7 wt % Cl^−^ at 70 °C: (**a**) WBE1, HAl77-2/316L SS/TA2; (**b**) WBE2, HAl-77-2/TA2/316L SS; and (**c**) WBE3, TA2/HAl77-2/316L SS.

Figure 21 shows the spatial potential and current–density distribution maps of three electrodes after being short-circuited for 12 h in artificial seawater with 2.7 wt % Cl^−^ at 70 °C. Regardless of changes in the sequence, the local potential of HAl77-2 is always the most negative, and its corresponding current is positive, indicating that HAl77-2 is a corrosion anode relative to the other two metals in the three coupled systems.

**Figure 22:**
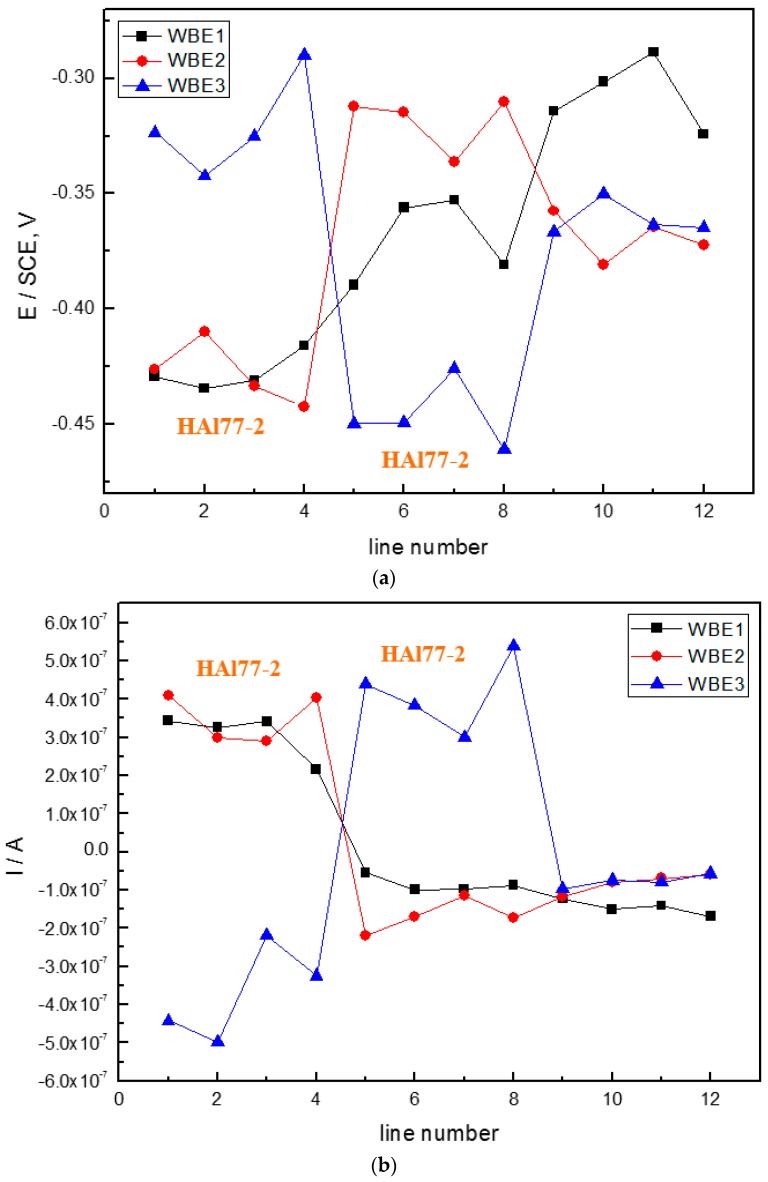
(**a**) Average potential; and (**b**) current–density distribution of each microelectrode line after being coupled for 12 h in artificial seawater with 2.7 wt % Cl^−^ at 70 °C.

Figure 22 shows the average potential and the current–density distributions of each microelectrode line after being short-circuited for 12 h in artificial seawater with 2.7 wt % Cl^−^ at 70 °C. In the coupling WBE1 system, when the anodic HAl77-2 wires are located at the edge and are connected to the 316L SS wires, the current value of the HAl77-2 wires decreases from the first-line to the fourth-line wires, and the fifth-line wires (316L SS) display anodic potential and current. As a result, the main anodic area enlarges and is even adjacent to the 316L SS wires near the HAl77-2. In the coupling WBE2 system, when the anodic HAl77-2 wires are also located at the edge and are connected with cathodic TA2 directly, the fourth-line (HAl77-2) higher current values are obtained, indicating a character of local anodic corrosion. The corrosion potential of HAl77-2 in WBE3 is the lowest, and the corrosion current density is the highest. Therefore, the corrosion of HAl77-2 is the most serious when the HAl77-2 is between 316L SS and TA2. In the corrosion cell, the electronic cathode can receive electrons from the anode and form a loop. When the anodic HAl77-2 is in the middle, the two cathode metals (TA2 and 316L SS) are evenly distributed around the anode. It has a shorter distance and less resistance between the cathode and the anode. Thus, the corrosion of HAl77-2 in WBE3 is more significant than in WBE1 and WBE2. Moreover, the current values of the fifth and eighth lines in WBE3 are significantly higher than those in the sixth and seventh lines. This shows that corrosion at the TA2/HAl77-2 and HAl77-2/316L interfaces are more serious than if they were away from the interface.

## 4. Conclusions

Since the surface area of each wire in the WBE is much smaller compared to the total electrode working area, each wire surface can be assumed to be electrochemically uniform even if the whole WBE surface is electrochemically non-uniform. This assumption allows electrochemical theories and methods of describing uniform electroplating and electrodissolution processes to apply to each wire in a WBE. It is possible to obtain not only the average electrochemical signal but also the localized electrochemical signals from the WBEs. In addition, WBEs should equally consist of an electrode matrix of different metal or alloy wires. Clearly, WBEs could be ideally suited for investigating the galvanic corrosion and localized corrosion of different metals or alloys couples. Our observations above led to several conclusions:

(1) The potential of HAl77-2 is lowest. Its corresponding local current density is the most positive of the three metals. The potential and current distribution of 316L SS was between the values of the HAl77-2 and the TA2, accompanied by numerical fluctuations. HAl77-2 is always the anode in these three-metal coupled systems. The TA2 is the cathode. Some 316L SS wires are cathodes, and the others are local anodes.
(2) The potential of the three metals decreases with increasing temperature. The current of HAl77-2 increases with temperature indicating enhanced anode corrosion at elevated temperature.
(3) Geometrical arrangement is an impact factor for the corrosion of the three-material coupled system. When the anodic HAl77-2 wires are located at the edge and are connected to the 316L SS wires, the main anodic area enlarges and is even adjacent to the 316L SS wires near the HAl77-2. When the anodic material HAl77-2 wires are located between the other two materials, the most severe corrosion occurs compared with the other two geometrical arrangements. Corrosion of the anode is the most significant when the HAl77-2 is in the middle of the coupled system. Corrosion at the TA2/HAl77-2 and HAl77-2/316L interface become more serious father from the interface.

## Figures and Tables

**Table 1:** Composition of HAl77-2, 316L SS, and TA2.

**HAl77-2**	**Composition**	**Cu**	**C**	**Zn**							
	wt %	59.33	0.93	39.74							
**316L SS**	**Composition**	**C**	**Si**	**Mn**	**S**	**P**	**Ni**	**Mo**	**Cr**	**Cu**	**Fe**
	wt %	0.02	0.33	1.5	0.02	0.029	10.1	2.1	16.7	0.4	68.80
**TA2**	**Composition**	**C**	**Si**	**Ti**							
	wt %	0.26	1.06	98.68							

## Supplementary Materials

The following are available online at http://www.mdpi.com/1996-1944/11/3/357/s1, Figure S1: OCP (vs. SCE/V) distribution maps of the WBE1 after immersion in artificial seawater at different temperatures: (a) 30 °C; (b) 40 °C; (c) 50 °C; (d) 60 °C; and (e) 70 °C, Figure S2: Spatial potential (left, E vs. SCE/V) and current–density (right, I/A) distribution maps of WBE1 after being short-circuited for 12 h in artificial seawater at different temperatures: (a) 30 °C; (b) 40 °C; (c) 50 °C; (d) 60 °C; and (e) 70 °C.
