# Supplementary material for: Mapping the Galvanic Corrosion of Three Metals Coupled with a Wire Beam Electrode: The Influence of Temperature and Relative Geometrical Position
Source: Materials (Basel). 2018 Feb 28;11(3):357. doi: 10.3390/ma11030357 (PMC5872936; doi:10.3390/ma11030357)
Supplement: Supplementary file 1 [file materials-11-00357-s001.pdf]

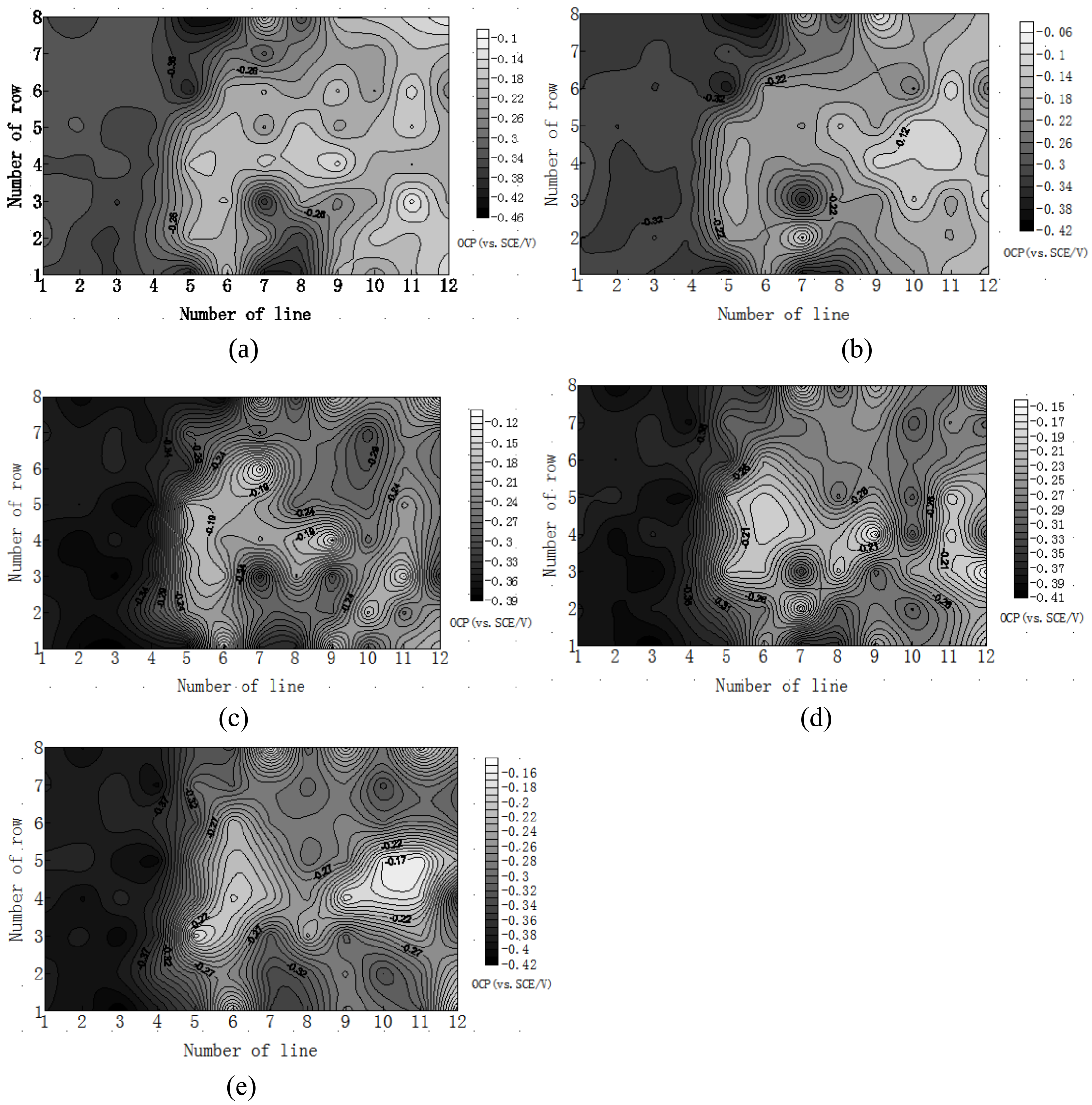


Figure S1. OCP (vs. SCE/V) distribution maps of the WBE1 after immersion in artificial seawater at different temperatures: (a) 30°C; (b) 40°C; (c) 50°C; (d) 60°C; (e) 70°C.

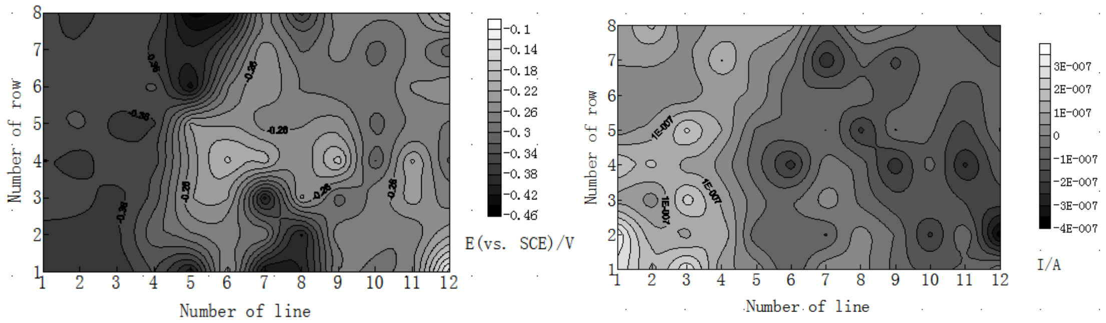
Number of row
Number of line
-0.1
-0.14
-0.18
-0.22
-0.26
-0.3
-0.34
-0.38
-0.42
-0.46
E(vs. SCE)/V
3E-007
2E-007
1E-007
0
-1E-007
-2E-007
-3E-007
-4E-007
I/A

(a)

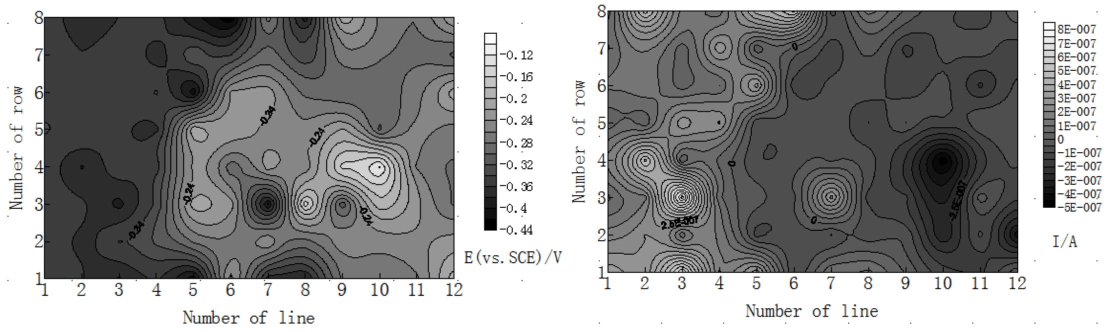
Number of row
Number of line
-0.12
-0.16
-0.2
-0.24
-0.28
-0.32
-0.36
-0.4
-0.44
E(vs. SCE)/V
8E-007
7E-007
6E-007
5E-007
4E-007
3E-007
2E-007
1E-007
0
-1E-007
-2E-007
-3E-007
-4E-007
-5E-007
I/A

(b)

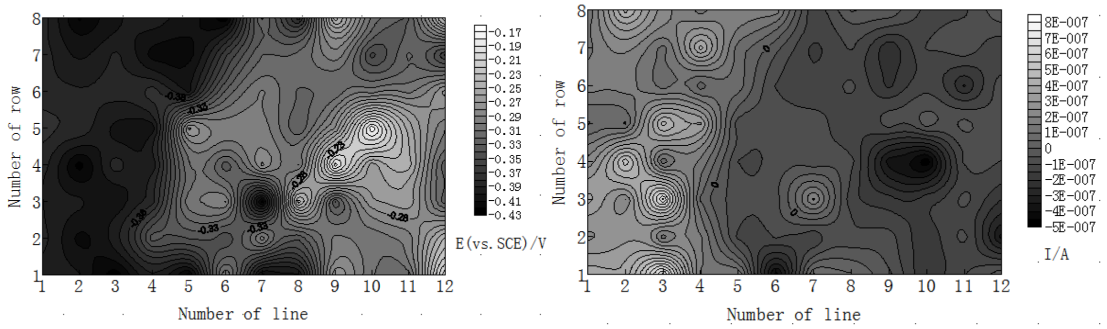
Number of row
Number of line
-0.17
-0.19
-0.21
-0.23
-0.25
-0.27
-0.29
-0.31
-0.33
-0.35
-0.37
-0.39
-0.41
-0.43
E(vs. SCE)/V
8E-007
7E-007
6E-007
5E-007
4E-007
3E-007
2E-007
1E-007
0
-1E-007
-2E-007
-3E-007
-4E-007
-5E-007
I/A

(c)

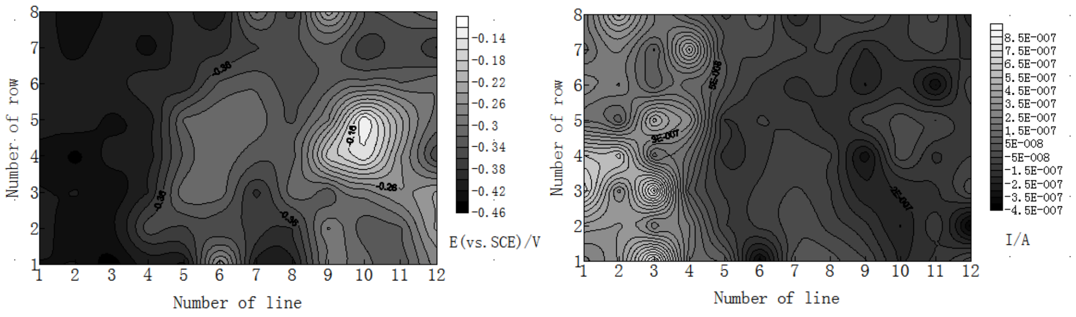
Number of row
Number of line
-0.14
-0.18
-0.22
-0.26
-0.3
-0.34
-0.38
-0.42
-0.46
E(vs. SCE)/V
8. 5E-007
7. 5E-007
6. 5E-007
5. 5E-007
4. 5E-007
3. 5E-007
2. 5E-007
1. 5E-007
5E-008
-5E-008
-1. 5E-007
-2. 5E-007
-3. 5E-007
-4. 5E-007
I/A

(d)

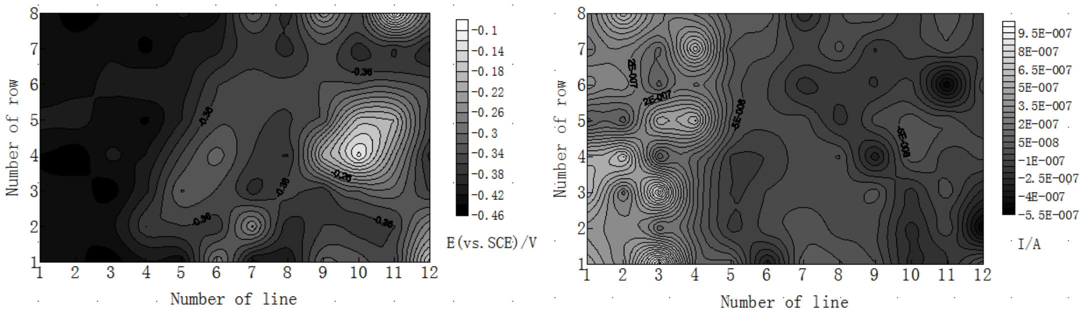
Number of row
Number of line
-0.1
-0.14
-0.18
-0.22
-0.26
-0.3
-0.34
-0.38
-0.42
-0.46
E(vs. SCE)/V
9. 5E-007
8E-007
6. 5E-007
5E-007
3. 5E-007
2E-007
5E-008
-1E-007
-2. 5E-007
-4E-007
-5. 5E-007
I/A

(e)

Figure S2. Spatial potential (left, E vs. SCE/V) and current-density (right, I/A) distribution maps of WBE1 after being short-circuited for 12 h in artificial seawater at different temperatures: (a) 30°C; (b) 40°C; (c) 50°C; (d) 60°C; (e) 70°C.
